# Comparison of two methods of measuring physical activity and its related components among middle age women: Pedometer versus self-report

**DOI:** 10.1371/journal.pone.0319288

**Published:** 2025-02-26

**Authors:** Mohammadreza Ghaneapur, Mohamad Ezati Asar, Marzieh Belji Kangarlou, Elahe Saleh

**Affiliations:** 1 Department of Public Health, Damghan school of public Health, Semnan University of Medical Sciences, Semnan, Iran; 2 Social Determinants of Health Research Center, Semnan University of Medical Sciences, Semnan, Iran; 3 Department of Occupational Health and Safety, Faculty of Medical Sciences, Tehran University of Medical Science, Tehran, Iran; 4 Student Research Committee, Semnan University of Medical Sciences, Semnan, Iran.; Karolinska Institutet, SWEDEN

## Abstract

**Introduction:**

Physical activity is essential for health maintenance across various dimensions; however, sedentary behavior remains widespread in numerous societies. Notably, women often exhibit higher levels of sedentary behavior than men, potentially exacerbating complications related to motherhood. This study aims to assess and compare physical activity levels in middle-aged women (30–45) using self-reported measures and pedometers while exploring associated factors in the context of reproductive health.

**Methods:**

This study analyzed secondary data from a randomized controlled trial involving 91 women aged 30–45 from Semnan University of Medical Sciences. Participants completed a validated 12-item questionnaire and wore Omron Style Pro-2 pedometers for one week to assess physical activity levels. Women were categorized as active or inactive based on self-reports and pedometer data. Statistical analyses were conducted using SPSS version 27. Participants provided informed consent, ensuring confidentiality throughout the study.

**Results:**

The study involved 91 women with a mean age of 27.4 years, of whom 82.6% were married. Only 11.7% self-reported as “low active,” while pedometer data revealed that 94.5% were inactive or had insufficient physical activity. A significant relationship was found between the number of children and physical activity levels, with women who had children being more active (p =  0.035). No correlation was observed between self-reported activity and pedometer measurements, indicating women often overestimate their physical activity levels.

**Discussion:**

This study highlights the inaccuracies in self-reported physical activity assessments. Most women were found to be physically inactive, with the number of children influencing their activity levels. These findings underscore the need for interventions to increase women’s physical activity.

**Conclusion:**

These findings bear important implications for health policy within communities. Further research involving larger sample sizes across various age and gender demographics is warranted to enhance our understanding of physical activity patterns and inform targeted interventions.

## 1. Introduction

Physical activity plays a significant role in an individual’s physical and mental health. As Professor Jeremy Morris stated over 25 years ago, the World Health Organization’s guidelines (2020) emphasize that investment in physical activity (PA) remains the “best buy” for public health [1]. Recent estimates indicate that supporting individuals to engage in more physical activity could prevent approximately 3.5 million global deaths annually [2]. According to available evidence, physical inactivity is identified as the fourth leading global cause of mortality (6% of all deaths), and insufficient activity is estimated to be the primary cause of 21–25% of breast and colon cancer cases, 27% of diabetes cases, and nearly 30% of the burden of ischemic heart diseases [3]. In other words, approximately 75% of healthcare costs are attributed to chronic diseases resulting from unhealthy behaviors such as tobacco use and exposure, malnutrition and food insecurity, and physical inactivity [4]. Based on existing definitions, any type of bodily movement created by skeletal muscles that requires energy expenditure is considered “physical activity.” Physical activity is a multidimensional behavior and does not occur in vacuum conditions, so its definition should encompass several related structures such as sedentary behavior, energy expenditure, and physical readiness [5].

Based on “Global Strategy on Diet, Physical Activity, and Health”, “physical activity” was identified as a key determinant of overall energy expenditure, serving as the basis for energy balance and weight control [6]. Despite the proven role and importance of physical activity in promoting optimal health throughout one’s life [7], study results indicate that in the United States, only one in five adults engaged in an acceptable level of physical activity [8], and the awareness of individuals about the existence of government guidelines for physical activity in 2008 was 36.1%, with only 1% having knowledge about the guidelines for engaging in moderate-intensity physical activity [9]. In Iran, physical inactivity is prevalent, especially among women and the elderly. This issue is so serious that 40% of Iranian adults (31% of men and 48% of women) are classified as insufficiently active.

Self-report questionnaires, interviews, and objective tools such as pedometers and accelerometers are used to assess individuals’ levels of physical activity [10]. However, health guidelines and disease-related recommendations regarding physical activity are based on data reported by individuals themselves rather than by tools and devices [11]. However, the results of some studies have shown that self-report methods do not show an accurate estimate of the absolute values of physical activity, and there is limited empirical evidence in this regard [12]. During reproductive years, the conditions of being overweight and physical activity differ and require increased attention, as women are at a greater risk for weight gain and obesity during this period. Women aged 18 to 50 have a higher likelihood of gaining weight compared to women over the age of 50 [13]. The reproductive period, which includes the time before, during, and after childbirth, is a crucial phase that is associated with weight gain and fat accumulation in mothers [14]. The importance of this issue is highlighted by the fact that after childbirth, women, on average, experience an increase in weight of 500 to 3000 grams [15]. The appropriate weight of a mother during this period is significant because having excess weight and obesity prior to and during pregnancy can increase maternal-related complications and unfavorable outcomes during delivery [16]. Findings from a study conducted by Awoke and colleagues regarding physical activity levels during the reproductive period indicate that physical activity among women significantly decreases during pregnancy. Accordingly, the percentage of women adhering to physical activity guidelines (total activity in minutes) among pregnant women (31.0%) was lower than that of women before pregnancy (59.4%) and women after childbirth (48.8%) [17]. Additionally, other studies have shown that 80% of pregnant women engage in insufficient physical activity, and this situation persists even after childbirth [18]. Given that being overweight among women is often viewed as a major concern for their health or body image, increasing physical activity, along with reducing caloric intake, is often recommended to help women lose weight [19]. In many global studies and those conducted in Iran, the age range of middle-aged women has been considered to be between 30 and 50 years. The findings also revealed that 12.2% of women were in a menopausal state [20]. The results of a study conducted by Ranjbar and colleagues on 17,178 Iranian women aged 20–40 also indicated that, on average, there is a gap of 15.4 ± 0.2 months between marriage and the first pregnancy for women [21]. Findings from studies conducted in Iran by Golshiri and colleagues on 960 women in menopause regarding menopause-related factors indicated that the average age of natural menopause among the studied Iranian women was 48.66 ± 3.79 years [22]. Considering the likelihood of menopause in women—as a confounding variable—and taking into account the results of the mentioned studies, this research focused on three 5-year age groups, specifically women aged 30 to 45, as the target age group. Therefore, considering the prevailing social and cultural conditions in Iran, this study was conducted with the aim of examining and comparing the physical activity of middle Age women (30–45 years), using both self-reporting and pedometer-based methods, as well as exploring related components.

## 2. Materials and methods

Preliminary data were obtained from a study based on Self-Determination Theory (SDT) in the community context regarding physical activity among women aged 30–45 years covered by Semnan University of Medical Sciences, Iran. The methodology and full details of the mentioned protocol have been published elsewhere [23].

### 2.1. Participants

In this study, a portion of the data from the research conducted as an RCT on 91 women aged between 30 and 45 years old in selected urban health comprehensive centers in Damghan, Semnan University of Medical Sciences, has been evaluated and analyzed “secondarily”. All women aged 30–45 were considered as the statistical population, and among the women who were eligible and willing to participate in the research, 91 women were randomly included in the study and were evaluated. In this study, women aged 30 to 45 years, BMI ≤ 35 were considered as inclusion criteria, and the presence of medical prohibition to do physical activity and being pregnant were among the exclusion criteria.

### 2.2. Measuring physical activity

Ninety-one middle-aged women aged 30–45 participated in the study, completing a valid and reliable 12-item questionnaire and using the Omron Style Pro-2 pedometer/accelerometer for one week. Based on the data derived from the questionnaires, participants were divided into active and inactive groups, and according to the data collected by the pedometers, women were categorized into sufficient and insufficient activity levels. Respondents who engaged in vigorous activity for at least 20 minutes a day for 3 days or more, or moderate-intensity activity and walking for at least 30 minutes a day for 5 days or more, or more than 5 days of any combination of walking, moderate-intensity activities, and vigorous activity for at least 3 days a week, or 7 days or more of any type of activities such as walking, at moderate or vigorous intensity, were considered “active.” Respondents who did not meet these criteria were classified as “inactive” [24].

### 2.3. A- Assessment of physical activity through self-report method

All eligible women completed a physical activity assessment questionnaire, which has been previously evaluated for its validity and reliability [25,26]. Twelve questions were selected from the available items in the initial study checklist. These included factors such as age, marital status, years of marriage, number of children, individual and spouse’s education level, employment status, work history, access to green spaces, membership in a mosque, organization, or social group as some potential components related to engaging in physical activity were examined. Additionally, the women’s daily and weekly physical activity levels were assessed through self-report by answering the following questions:

A- How many sessions of physical activity do you usually engage in each week?
B- How many minutes of exercise do you typically perform in each session?

### 2.4. B- Measurement of physical activity by pedometer method

In this study, all participants were familiarized with and accepted the use of a pedometer, and were instructed to wear it as a necklace at all times of the day (except during bathing). To reduce potential irritation from wearing the pedometer, individuals were provided with this tool for a period of 3 weeks. However, the physical activity levels of the women were assessed in the third week. In order to minimize potential errors in transferring and calculating the physical activity data of each woman, Omron walking style Pro 2.0 pedometers/accelerometers (made in China) equipped with USB and the ability to connect to a computer were used. This specific pedometer is one of the most professional pedometers with three-dimensional physical activity sensors, which can easily be placed in a pocket, on a belt, or worn as a necklace on the individual’s body to calculate the number of steps, distance traveled, aerobic steps, and calories burned through walking. To calibrate the pedometer, the average step length of each individual was calculated by taking 10 consecutive steps. In each pedometer setup, the individual’s height, weight, and calculated average step length data were recorded in the pedometer settings. Although acquiring this pedometer/accelerometer with specific specifications was challenging under sanctions, it has other advantages: this pedometer only records the individual’s physical activity based on input data and does not register other movements of the device caused by shaking or even relocating it by the user. After connecting each pedometer to a computer, the recorded data from each pedometer was extracted using the Bi-LINK Gateway 2.2.0.0 software (free access for academic purposes).

### 2.5. Statistical methods/ sample size

As stated, this study is part of a clinical trial. Additionally, according to the study by Murphy et al. [27], the correlation coefficients between the three self-report questionnaires and the objective measurement tool for physical activity (accelerometer) were calculated as 44.5%, 45.2%, and 77.4%, respectively. To achieve a power of 0.8 and considering an α = 0.05 and an expected difference of 0.25, the sample size was determined to be 59 individuals using Stata software. However, in the present study, 91 individuals were examined as the sample. In other cases, data were analyzed using SPSS version 27. This command calculates the power assuming a correlation coefficient of 0.44 and a difference of 0.25. The normality of quantitative data was measured using the Kolmogorov-Smirnov test, and according to the results obtained from the Kolmogorov-Smirnov test, T-test or Mann-Whitney test was used to compare two groups, and more than three groups were compared using analysis of variance or Kruskal-Walli’s test. To assess the correlation between self-reported data and the values measured by the pedometer, spearman correlation was utilized. Descriptive statistics were also used to report the percentage, mean, and standard deviation. To address the issue of multiple comparisons, Bonferroni correction was used, and the level of significance was adjusted according to the number of comparisons.

### 2.6. Ethical considerations

This project and all related appendices (and the informed consent form) have been approved by the Ethics Committee of the Technology Research Assistant of Tehran University of Medical Sciences (Ethical Code: IR.TUMS.REC.1394.1020]. Also, the protocol designed and registered by Ghaneapur et al. has been approved in Iran Clinical Trials Registration Organization (IRCT2016020223072N1). Therefore, the current study is based on a part of the secondary data related to the large study conducted (between May 2017 and August 2019) [25, 26]. Research investigator briefed participants about the study and asked them to answer all the items in the questionnaires. Participation in the study was voluntary and women signed of an informed written consent form. Moreover, before collecting data participants were ensured of the confidentiality of their personal data and the relevant ethical aspects. After conducting the study, the data related to physical activity and relevant analyses were made available to each participant.

## 3. Results

The mean age of the women participating in this study was 27.4 ± 2.36 years. 82.6% of the women were married, and on average, there were 18 children per 10 women. 46.7% of the women had university education, and only 9.23% of them had administrative jobs. 43.7% of them were members of social networks (such as mosques, organizations, or public interest groups), and 64% had access to parks and green spaces. In this study, the number of aerobic steps, total steps, and related components were examined and compared (Table 1).

**Table 1:** Physical activity of participants separated by demographic and background variables.

Pedometer				self-reporting	
Variable Number	Number of steps (aerobic and non-aerobic)	Number of aerobic steps (Mean ± SD)	*P-Value* Number of steps (Number of aerobic steps)	Physical activity time in the day (Mean ± SD)	*P-Value*
	(Mean ± SD)	(Mean ± SD)		(Mean ± SD)	
**Marital status**					
Married (76)	4687.57 ± 237.45	1146.28 ± 214.24	0.093 (0.341)	50.9 ± 28.81	0.215
Single (12)	3624.5 ± 490.86	622.67 ± 247.68		63.9 ± 31.6	
**Number of Children**					
No child(14)	3108 ± 432.81	418.14 ± 211.35	0.011 (0.035)	63.33 ± 32.5	0.758
One(58)	4827.58 ± 279	1338.16 ± 270.22		43.03 ± 18.40	
More than two (19)	4607.9 ± 369.95	620.63 ± 196.7		76.25 ± 40.18	
**Education**					
Non-university degree (45)	4339 ± 303.77	1281.32 ± 316.66	0.352 (0.268)	60.16 ± 30.99	0.036
University (43)	4747.58 ± 314.13	861.98 ± 199.85		44.82 ± 25.59	
**Employment status**					
Employed (22)	4799.45 ± 436.69	821.68 ± 180.53	0.494 (0.439)	58.33 ± 37.42	0.754
Housewife (66)	4453.45 ± 253.04	1159.48 ± 244.53		50.49 ± 25.19	
**Access to a green area**					
Yes (57)	4602.77 ± 267.88	1043.04 ± 233.03	0.769 (0.764)	51.54 ± 30.93	0.496
No (31)	4467.77 ± 382.05	1161.68 ± 322.93		54.17 ± 26.25	
**Social media user**					
Yes (38)	4117.79 ± 303.01	936.76 ± 299.95	0.083 (0.502)	57.34 ± 30.56	0.173
No (49)	4884.81 ± 206.02	1195.67 ± 245.95		47.22 ± 27.78	

The values obtained from the assessment of women’s physical activity by pedometer were compared with the WHO’s recommended values. The findings of this study indicated that a total of 94.5% of the examined women were inactive or had insufficient physical activity. The data on physical activity reported by self-assessment and measured by pedometer/accelerometer were compared. Out of the 91 women participating in the study, only 60 accurately reported their physical activity. Based on the data collected on physical activity through self-reporting, only 11.7% of women classified themselves as having “low activity,” and in 3.88% of cases, they reported their physical activity level as “sufficiently active.” (Table 2).

**Table 2:** Physical activity of participants measured using self-reported data and pedometer.

Inactive or insufficient physical activity (measured by a pedometer)			Appropriate physical activity (measured by a pedometer)	
**Inactive**	**Insufficient**	**To somehow**	**Active**	**Highly active**
**Freq (%)**	**Freq (%)**	**Freq (%)**	**Freq (%)**	**Freq (%)**
54 (59.3)	32 (35.2)	3 (3.3)	2 (2.2)	0 (0)
**Inactive or insufficient physical activity** **(**measured using self-reported data**)**			**Appropriate physical activity** **(**measured using self-reported data**)**	
**Freq (%)**			**Freq (%)**	
53 (88.3)			7 (11.7)	

There was no correlation observed between the amount of physical activity reported by the women and the number of steps measured by the pedometer; in other words, the women did not accurately estimate their physical activity levels (r = 0.037, p = 0.779) (Table 3).

**Table 3:** Correlation observed between self-reported and pedometer.

Duration of exercise per week		Number of aerobic steps	Number of steps (aerobic and non-aerobic)
Correlation Coefficient	0.152	0.037
p-value	0.247	0.779
N	60	60

The physical activity measured by the pedometer was analyzed based on each of the demographic and social components. There was almost no significant relationship observed between most of the mentioned components and the level of physical activity of women, except for a significant relationship between the number of children and the level of physical activity (p-value = 0.035), it was found that women with children engaged in a higher level of physical activity compare to those without children (Table 1). The relationship between the number of children and physical activity was examined while considering Bonferroni correction and the significance level (0.017 at α = 0.05). A significant relationship was found between the number of children and physical activity (p-value = 0.011) (Table 1).

## 4. Discussion

Based on the self-reported data in this study, only 11.7% of the women in the study identified themselves as “sedentary or low-active,” but by measuring the steps recorded by pedometers, it was found that 94.5% of women in the “sedentary and low-active” group met the recommended number of steps by the World Health Organization [28]. The results of the upcoming studies are largely similar to the findings of the study by Mohamad Hasnan Ahmad and colleagues. In their study, all 169 women participating in the research were classified into the active group based on self-reported data, whereas only 8.4% of those same women were categorized as having sufficient physical activity based on the data recorded by the pedometer. Another similarity between the two studies is that women used the pedometer for one week. However, in our study, the pedometer was provided to all women, while in the aforementioned study, only 63.3% of the women used the pedometer [29]. In a study conducted by Jahan and colleagues, it was also found that in self-reporting method, only 55% of women and 44% of middle-aged men considered themselves as low-active. However, in self-reported data or step counts measured by pedometers, it was revealed that 80% of respondents were low-active and the number of women exceeded that of men [30]. In a study conducted in Rasht, it was also found that only 28.4% of women engaged in vigorous physical activity [31], although in the present study, the intensity of physical activity was not the focus of the researchers. The components related to physical activity were not examined in the mentioned study, and since the comparison of step counts in men was not the focus of our study, we are unable to make comparisons in this regard. The findings of this research align to a large extent with the results of the study by Hosseini and colleagues, who reported insufficient physical activity in 75% of middle-aged women in a consistent manner [32]. Furthermore, the results of the present study show some similarities with the findings of another study that reported a 69% rate of physical inactivity among women in Ardabil [33]. Based on the studied components, this research only observed a significant relationship between the number of children and the level of physical activity. However, in a study conducted by Hosseini and colleagues, an inverse relationship was reported between the age of women and the duration of intense and moderate physical activity, and a direct relationship was reported between the individual’s level of education and the duration of intense and moderate physical activity [32]. The results of studies conducted in other cities in Iran (such as Rasht) also indicate that only 28.4% of women engage in intense physical activity [31]. Looking at the results of studies in other regions of the world, a more favorable situation is not observed, with rates of physical inactivity in Europe reported to be between 43.3% and 87.8% [34], and up to 80% of American adults are also reported to be in an undesirable state in terms of physical activity [35]. Referring to the findings of the present study and similar cases, it becomes evident that participants in such studies tend to self-report more physical activity. The results of this investigation align with a study conducted by Murphy and colleagues, which aimed to examine the validity and reliability of three methods for assessing and reporting physical activity among students.

The study by Murphy revealed weak to moderate correlations (r =  0.29–0.37, p < 0.01) between self-reported moderate to vigorous physical activity (MVPA) levels and accelerometer data in terms of minutes per day of physical activity, and these correlations (r = 0.29–0.47, p < 0.01) were particularly significant for women [36]. Overestimation of the number of steps reported by women participating in this study showed similarities with the findings of the study by Ekalak. In the study by Ekalak and colleagues, it was also found that self-reported physical activity results may lead to inaccurate determination of physical activity levels and may bias the relationship between physical activity and outcomes of interest [37]. Although the research by Joseph and colleagues identified family and caregiving responsibilities as one of the barriers to women’s participation in physical activities, it was noted that individuals without children have more leisure time compared to women with children [38], our study findings clearly indicate a significant relationship between having children and the number of steps taken by an individual, such that having children motivates individuals to engage in more physical activity. However, further evidence and reasons regarding this matter were not examined.

In the study conducted by Jahan and colleagues, the relationship between daily physical activity (measured as steps per day using pedometers and self-reporting methods) and health indicators was examined among 145 Indian individuals (n = 76 women and n = 69 men) aged 40 to 60 years [30]. In the mentioned study, the OMRON pedometer (HJ-325), which records the number of steps taken throughout a day, was used for only 3 consecutive days. The reading and recording of the number of steps each day, as well as resetting the pedometers, were the responsibility of the participants themselves. The average number of steps per day for women was 1558 ± 3226 and for men was 2530 ± 4273. A positive and significant relationship was found between the number of steps measured by the pedometer and the self-reported physical activity (r = 0.5, p   <  0.001) [30]. However, compared to the aforementioned study, utilizing more advanced pedometers with the capability of automatically storing and transferring data via USB, which eliminates the need for individual intervention and daily manual adjustments, the measurement of step counts over a longer duration (7 days) and having a larger sample size are among the advantages of our study design. While Jahan and colleagues reported a significant correlation between the steps measured by the pedometer and the steps reported by individuals, no such correlation was observed in our study. The findings from both studies indicated that the majority of the middle-aged individuals surveyed were sedentary.

## Conclusions

The complexity of the relationship between physical activity, individual health status, and social functioning has been emphasized in many studies. In many of these studies, single-axis accelerometers have been used, which are often placed on limited areas of the body such as the hip and do not assess upper body activities or restricted hip movements in activities like stationary cycling or weight lifting effectively [39], but in this study 3-axis pedometers/accelerometers with higher accuracy were utilized, which only count the number of steps taken by the individual without registering other movements. Additionally, the use of this type of pedometer, which was available in the form of a necklace and did not restrict the participants’ other activities, was well-received by the women involved in the study. Additionally, considering the potential variation in physical activity on different days of the week, researchers focused on the average number of steps taken by individuals over the course of a week. The findings of this study indicated that the assessment of physical activities based solely on questionnaires and subjective methods is unreliable and should not serve as the basis for judgment and evaluation, thereby recommending the use of objective methods. Furthermore, increasing the number of study samples from various age and gender groups is recommended. Patterns and predictors of physical activity are clearly influenced by gender and other factors, and the benefits, barriers, and psychological consequences of physical activity for women, especially in cases of communication difficulties due to issues such as urinary incontinence, depression, mood disorders, and obesity [40], are among the issues that should be considered in future studies. Although assessing individuals’ physical activity is limited by self-reporting methods and has its own constraints and ambiguities, the main challenge when comparing data collected by accelerometers and questionnaires is that these two methods measure related but dissimilar constructs. This means that accelerometers measure the accelerations of movements in physical activities, and such instruments do not directly measure an individual’s behaviour [39]. Despite the identified limitations and ambiguities, the self-reporting method for assessing physical activity continues to be utilized and is often used as a basis for judgment [41–43]. While the dose-response relationship of physical activity for preventing non-communicable diseases in healthy individuals in specific age groups has been endorsed [44], noticeable sedentary behavior and concerns of women as valuable family members on one hand, and the lack of credibility of mental and self-report methods in assessing physical activities and other health measurements on the other hand, highlight the need for changes in assessment methods and decision-making in health-related areas. Therefore, the adaptation of physical activity enhancement programs for women considering cultural backgrounds and in a more social context is necessary. Additionally, the study highlights that reliance solely on questionnaires and subjective methods for assessing physical activity is unreliable and should not serve as the basis for judgment and evaluation; thus, the use of objective assessment methods is strongly advised.

### Strengths

The study collected information on physical activity levels through self-reporting and standard pedometers, providing detailed and accurate data. The use of three-dimensional accelerometers captured all physical activities of the studied women throughout the day, offering a comprehensive assessment. The study utilized the Omron Style Pro 2 3D pedometer/accelerometer and software for data extraction, ensuring accurate measurement of physical activities.

### Limitations

The study had several limitations, including its reliance on self-reported physical activity data, which can be subjective and prone to overestimation. The sample size of 91 women may limit the generalizability of the findings. Additionally, the study focused on a specific geographic region, which may not reflect habits in other areas. Lastly, the duration of using pedometers for just one week might not capture long-term activity patterns. These factors could impact the robustness of the results and their applicability to a broader population. Since the duration and intensity of diverse physical activities of the individuals were not a focus for the researchers, there was no possibility of standardizing and converting the physical activity of women into comparable quantitative units, such as Metabolic Equivalent minutes per week (MET-min/week).

## Abbreviations

PA: Physical Activity
BMI: Body Mass Index
WHO: World Health Organization
